# A phylogenetic study of dengue virus in urban Vietnam shows long-term persistence of endemic strains

**DOI:** 10.1093/ve/vead012

**Published:** 2023-02-16

**Authors:** James Ashall, Sonal Shah, Joseph R Biggs, Jui-Ning R Chang, Yalda Jafari, Oliver J Brady, Huynh Kim Mai, Le Thuy Lien, Hung Do Thai, Hien Anh Thi Nguyen, Dang Duc Anh, Chihiro Iwasaki, Noriko Kitamura, Marnix Van Loock, Guillermo Herrera-Taracena, Freya Rasschaert, Liesbeth Van Wesenbeeck, Lay-Myint Yoshida, Julius Clemence R Hafalla, Stephane Hue, Martin L Hibberd

**Affiliations:** Department of Infection Biology, Faculty of Infectious and Tropical Diseases, London School of Hygiene and Tropical Medicine, Keppel Street, London, WC1E 7HT, UK; Department of Infection Biology, Faculty of Infectious and Tropical Diseases, London School of Hygiene and Tropical Medicine, Keppel Street, London, WC1E 7HT, UK; Department of Infectious Disease Epidemiology, Faculty of Epidemiology and Population Health, London School of Hygiene and Tropical Medicine, Keppel Street, London, WC1E 7HT, UK; Department of Infection Biology, Faculty of Infectious and Tropical Diseases, London School of Hygiene and Tropical Medicine, Keppel Street, London, WC1E 7HT, UK; Department of Infectious Disease Epidemiology, Faculty of Epidemiology and Population Health, London School of Hygiene and Tropical Medicine, Keppel Street, London, WC1E 7HT, UK; Department of Infectious Disease Epidemiology, Faculty of Epidemiology and Population Health, London School of Hygiene and Tropical Medicine, Keppel Street, London, WC1E 7HT, UK; Centre for the Mathematical Modelling of Infectious Diseases, London School of Hygiene and Tropical Medicine, Keppel Street, London, WC1E 7HT, UK; Department of Microbiology and Immunology, Pasteur Institute of Nha Trang, Xương Huân, Nha Trang, 650000, Vietnam; Department of Microbiology and Immunology, Pasteur Institute of Nha Trang, Xương Huân, Nha Trang, 650000, Vietnam; Department of Microbiology and Immunology, Pasteur Institute of Nha Trang, Xương Huân, Nha Trang, 650000, Vietnam; National Institute of Hygiene and Epidemiology, 1 P. Yec Xanh, Phạm Đình Hổ, Hai Bà Trưng, Hà Nội, 100000, Vietnam; National Institute of Hygiene and Epidemiology, 1 P. Yec Xanh, Phạm Đình Hổ, Hai Bà Trưng, Hà Nội, 100000, Vietnam; Paediatric Infectious Diseases Department, Institute of Tropical Medicine, Nagasaki University, 1-12-4 Sakamoto, Nagasaki 852-8523, Japan; Department of Infectious Disease Epidemiology, Faculty of Epidemiology and Population Health, London School of Hygiene and Tropical Medicine, Keppel Street, London, WC1E 7HT, UK; Paediatric Infectious Diseases Department, Institute of Tropical Medicine, Nagasaki University, 1-12-4 Sakamoto, Nagasaki 852-8523, Japan; Janssen R&D, Janssen Pharmaceutica NV, Turnhoutseweg 30, Beerse B-2340, Belgium; Janssen Global Public Health, Janssen Research & Development, LLC, 800 Ridgeview Drive, Horsham, PA 19044, USA; Janssen R&D, Janssen Pharmaceutica NV, Turnhoutseweg 30, Beerse B-2340, Belgium; Janssen R&D, Janssen Pharmaceutica NV, Turnhoutseweg 30, Beerse B-2340, Belgium; Paediatric Infectious Diseases Department, Institute of Tropical Medicine, Nagasaki University, 1-12-4 Sakamoto, Nagasaki 852-8523, Japan; Department of Infection Biology, Faculty of Infectious and Tropical Diseases, London School of Hygiene and Tropical Medicine, Keppel Street, London, WC1E 7HT, UK; Department of Infectious Disease Epidemiology, Faculty of Epidemiology and Population Health, London School of Hygiene and Tropical Medicine, Keppel Street, London, WC1E 7HT, UK; Centre for the Mathematical Modelling of Infectious Diseases, London School of Hygiene and Tropical Medicine, Keppel Street, London, WC1E 7HT, UK; Department of Infection Biology, Faculty of Infectious and Tropical Diseases, London School of Hygiene and Tropical Medicine, Keppel Street, London, WC1E 7HT, UK

**Keywords:** virus, pransmission, persistence, dengue, phylogenetics, sequencing

## Abstract

Dengue virus (DENV) causes repeated outbreaks of disease in endemic areas, with patterns of local transmission strongly influenced by seasonality, importation via human movement, immunity, and vector control efforts. An understanding of how each of these interacts to enable endemic transmission (continual circulation of local virus strains) is largely unknown. There are times of the year when no cases are reported, often for extended periods of time, perhaps wrongly implying the successful eradication of a local strain from that area. Individuals who presented at a clinic or hospital in four communes in Nha Trang, Vietnam, were initially tested for DENV antigen presence. Enrolled positive individuals then had their corresponding household members invited to participate, and those who enrolled were tested for DENV. The presence of viral nucleic acid in all samples was confirmed using quantitative polymerase chain reaction, and positive samples were then whole-genome sequenced using an amplicon and target enrichment library preparation techniques and Illumina MiSeq sequencing technology. Generated consensus genome sequences were then analysed using phylogenetic tree reconstruction to categorise sequences into clades with a common ancestor, enabling investigations of both viral clade persistence and introductions. Hypothetical introduction dates were additionally assessed using a molecular clock model that calculated the time to the most recent common ancestor (TMRCA). We obtained 511 DENV whole-genome sequences covering four serotypes and more than ten distinct viral clades. For five of these clades, we had sufficient data to show that the same viral lineage persisted for at least several months. We noted that some clades persisted longer than others during the sampling time, and by comparison with other published sequences from elsewhere in Vietnam and around the world, we saw that at least two different viral lineages were introduced into the population during the study period (April 2017–2019). Next, by inferring the TMRCA from the construction of molecular clock phylogenies, we predicted that two of the viral lineages had been present in the study population for over a decade. We observed five viral lineages co-circulating in Nha Trang from three DENV serotypes, with two likely to have remained as uninterrupted transmission chains for a decade. This suggests clade cryptic persistence in the area, even during periods of low reported incidence.

## Introduction

Dengue virus (DENV) is one of the most important arthropod-borne viral infections in the world today, with roughly 2.5 billion people at risk of infection (Guzman et al. 2010), mainly in south-east Asia, South America, and the Caribbean. Globally, there are estimated to be 100–400 million new infections each year and around 40,000 deaths (Bhatt et al. 2013; Zeng et al. 2021). The number of confirmed infections reported to the World Health Organization has increased every year since 1990, primarily due to improvements in testing infrastructure and increased numbers of outbreaks, peaking in 2019 with 5.2 million cases and 4,032 deaths (World Health Organization [WHO] 2022).

DENV is an arthropod-borne virus of the *Flaviviridae* family that includes four genetically related yet antigenically distinct serotypes (D1, D2, D3, and D4). The virus is spread by the bite of *Aedes* mosquitoes, primarily *A. aegypti* (Cardoso et al. 2015), which are distributed in most tropical and subtropical areas of the world. Households have been proposed as the primary location for the insect vector (Stoddard et al. 2013), particularly those within areas of critical population density and without access to piped drinking water (Schmidt et al. 2011), with lower insect abundances associated with non-residential districts (Morrison et al. 2006). Vector control through prevention of biting, breeding site removal, and insecticide is currently the principal method of controlling DENV in endemic populations (Buhler et al. 2019). There are also new vector control measures, which are attempting to reduce or remove the vector population through the release of sterilised male mosquitoes (Alphey et al. 2010), as well as strategies that reduce the ability of mosquitoes to transmit the virus by introducing those infected with the *Wolbachia* bacteria (Utarini et al. 2021) that have had promising success in reducing reported DENV disease (Caragata et al. 2021). A vaccine for all DENV serotypes was developed in 2015 (Flasche et al. 2016) although a requirement for pretesting before use has limited its widespread adoption (Fongwen et al. 2021). There is also the possibility for antiviral treatment in the near future (Kaptein et al. 2021) that might have the potential for prophylactic intervention in outbreaks (Brady et al. 2021).

The persistence of DENV in a population is dependent upon several factors that involve the interplay between the human host and insect vector (Rico-Hesse 2010). The primary human factors are, therefore, population density and how many susceptible individuals remain or are added to a population as the virus spreads through it. This is particularly challenging to measure with DENV due to the complex cross-reactive role of antibodies between the four serotypes, associated with both immune protection and enhancement of infection (Nikin-Beers and Ciupe 2015; Screaton et al. 2015; Dejnirattisai et al. 2016). Mosquito vectors and the impact of the environment on their lifecycle are the primary reasons why DENV displays a seasonal pattern that is often closely related to rainfall and temperature, conditions vital for the development of the larval stages of their lifecycle (Yuan et al. 2020). Epidemiological data can indicate breaks in transmission during periods in which environmental conditions are unfavourable to mosquito breeding (Kyle and Harris 2008); yet because a high percentage of cases are asymptomatic and/or unreported, the virus could persist at levels below the threshold for detection (Ten Bosch et al. 2018; Biggs et al. 2021).

Previous studies have shown that locally circulating genotypes and strains from previous seasons are capable of contributing to seasonal spikes in case numbers and even outbreaks (Schreiber et al. 2009; Galarion et al. 2019; Takemura et al. 2022). However, the amount of time DENV serotypes and strains remain in an endemic population despite increasing immunity, control measures, and periods of very low circulation remains poorly studied. Additionally, the role of introduced strains in seasonal outbreaks against a background of highly endemic strains has not been extensively investigated.

The phylogenetic analysis of viral genomes enables the identification of closely related viruses in an infected population, with similar viral genetic identity being indicative of a recent transmission event between sampled cases (Campbell et al. 2018). This approach allows the determination of epidemiological linkage between observed infections without the reporting biases inherent to traditional contact tracing methods (Grubaugh et al. 2018b). DENV is a good candidate for this type of analysis, as it typically only infects a host for a very short amount of time (between 4 and 7 days in humans and up to the 1-month lifespan of the mosquito (Guzman et al. 2016)) before being transmitted to a new host, ensuring that the evolution dynamics, that is the evolutionary rate of the virus, are on a scale that closely correlates with transmission (Holmes 2004, 2009; Pybus, Fraser, and Rambaut 2013). Using these analyses, the transmission characteristics of DENV from an international scale can be inferred, thereby determining the likely origins of viral import that can have implications for control measures (Pollett et al. 2018).

Classifying viruses based on their genetic similarity using phylogenetic information gives us the most reliable metric of viral persistence in a population. Phylogenetic information coupled with molecular clock inference can additionally be used to determine the timing of epidemiological events along a phylogeny, such as the time to the most recent common ancestor (TMRCA) of a viral clade, which can be utilised to estimate a time of introduction of a particular strain or clade to a country or how long it has been in circulation in the area.

## Methods

### Study population

The study was conducted at the Nha Trang population-based cohort study site in central Vietnam. Enrolment of the study cohort was conducted in the communes of Vinh Hai, Vinh Phuoc, Vinh Tho, and Vinh Hoa in Nha Trang City, Vietnam, after local ethical review and approval from the National Institute of Hygiene and Epidemiology—Vietnam (study approval number IRB-VN01057). Individuals of all ages who resided in the selected communes and who visited either a local polyclinic (between October 2016 and May 2019) or the city’s Tropical Medicine Hospital (between December 2016 and April 2019) with suspected dengue were deemed eligible. In the polyclinic, those who presented with suspected dengue fever were approached, and those who consented to be enrolled in the study were subsequently tested for dengue with an NS1 rapid test (DENGUE NS1 AG STRIP, #70700, BioRad). In the hospital, only those who were diagnosed with dengue fever on the basis of an NS1 rapid test were enrolled in the study.

Study teams then visited the index case’s home addresses, where additional consenting members of the household were also enrolled and had blood samples taken. After consent was obtained, a questionnaire was completed by all enrolled individuals in order to gather information on clinical and demographic information relevant to this study, such as age, time of fever onset, home coordinates (using a Global Positioning System (GPS)), and typical daytime location.

Historical DENV case numbers were acquired from the Nha Trang Preventive Medicine Center, Khanh Hoa Center for Disease Control (CDC), and Tropical Diseases Hospital and were based on clinical signs and symptoms.

### DENV ribonucleic acid (RNA) detection

NS1-positive serum samples were processed using Qiagen viral RNA extraction kit and tested by real-time reverse-transcription quantitative polymerase chain reaction (RT-qPCR). Viral RNA detection and blood viral load quantification were determined using LightMix® Dengue Virus EC kit (Cat.-No. 58-0700-96, TIB MolBiol, Berlin, Germany), which can identify all four DENV serotypes, with Luna® Universal Probe One-Step RT-qPCR Kit (New England Biolabs, USA). RT-qPCR assay was then performed in Applied Biosystems® 7500 Fast Dx Real-Time PCR instrument (Thermo Fisher Scientific, USA) following cycling conditions in the manufacturer’s protocol.

The CDC real-time RT-qPCR assay was used to determine the serotype of DENV in the infected individuals. The protocol used was based on Santiago et al. (2013) with minor adjustments. The Ultraplex 1-Step Tough Mix qPCR kit (Quantabio) was substituted for the recommended kit with the manufacturer’s instructions followed.

### Sequence generation

RNA extracted from DENV quantitative polymerase chain reaction (qPCR)–positive patient sera was processed for whole-genome sequencing on the Illumina MiSeq platform. Two library preparation techniques were used; for samples that were positive by serotyping assay (to serotypes 1 or 2), an amplicon sequencing approach was used based on the protocol developed by Josh Quick for Zika virus (Quick et al. 2017). Briefly, multiple 400 basepairs amplicons were designed using the primal scheme software (available at http://primal.zibraproject.org/), which is based on primal3. Ten whole-genome sequences from temporally and spatially close outbreaks were used to design the sequencing primers.

For sequences with an unknown serotype but were positive for DENV RNA by the qPCR singleplex assay, a target enrichment approach was followed. Biotinylated probes with a length of 120 nucleotides (nt) were designed by running the Compact Aggregation of Targets for Comprehensive Hybridization programme (Metsky et al. 2019) on available DENV 1, 2, 3, 4 genomes from NCBI GenBank. The Agilent SureSelect^XT HS^ Target Enrichment kit and protocol was followed with the revisions recommended for pathogen sequencing (Williams et al. 2019).

Sequences generated in this study have been uploaded to NCBI GenBank with accession numbers OQ426566–OQ427062.

### Phylogenetic analysis

Consensus DENV full genomes of the target viruses were generated from the FASTQ files produced by Illumina MiSeq sequencing. Raw files were screened for quality and trimmed to remove primer sequence bias using Fastp (Chen et al. 2018). Cleaned FASTQ files were then aligned to a serotype-specific reference genome using the Burrows–Wheeler Alignment for short-reads (BWA-MEM) (Li 2009) along with the SAMtools view and sort packages (Li et al. 2009). Reference DENV genomes were extracted from GenBank: DENV1: JQ045626, DENV2: GU131898, and DENV4:NC_002640. Aligned reads were called using SAMtools mpileup and iVar consensus (Grubaugh et al. 2018a) programs with a resulting consensus genome sequence in FASTA format. The genotype and serotype of the sampled viral populations were confirmed from the generated consensus genomes, using the DENV typing tool available from the Genome Detective resource (Vilsker et al. 2019), which uses phylogenetics and pairwise distance within and between groups to known references to assign a genotype to a sequence.

Processed consensus genomes were aligned against all full-length DENV genomes available in the NCBI Virus Variation Resource Database (Hatcher et al. 2017) (as of 1 March 2022), per serotype, using MAFFT (Katoh and Standley 2013). The resulting alignments were visually checked and manually adjusted in AliView (Larsson 2014) to remove the 5’ and 3’ end untranslated regions (UTR), as these greatly differed in length between publicly available sequences and sequences that contained gaps where sequencing read depth (<200) was insufficient. Maximum-likelihood (ML) phylogenies were generated using IQTREE (Nguyen et al. 2015) under the best-fitting model of nucleotide substitution (as determined by the model finding procedure implemented in IQTREE) and 1,000 ultrafast bootstrap replicates for branch support assessment. The resulting trees were edited and mid-point rooted using FigTree.

Time-scaled phylogenies and root-to-tip linear regressions were inferred for each serotype with TreeTime (Sagulenko, Puller, and Neher 2018) using the ML trees generated with IQTREE. Time trees were estimated using the TreeTime function, with up to 500 interactions, taking into account covariation when estimating rates of nucleotide substitutions along the phylogeny and with automatic rerooting of the tree to maximise the clock-like signal. The ‘mugration’ model for ancestral state reconstruction implemented in TreeTime was used to infer the most likely migration patterns of the sampled viruses along the phylogeny, using the sampling location of the genomes as discrete traits (see Supplementary Table S1).

### Selection analysis

We investigated positive evolutionary pressure across the genomes of the two clades with the largest case numbers and which also spanned over the greatest time period (D1.I and D4.I) using the MEME (Murrell et al. 2012) and FUBAR (Murrell et al. 2012) tools from datamonkey.org (Weaver et al. 2018) to investigate the potential presence of episodic and pervasive selection pressures, respectively. The dengue polyprotein amino acid sites that the models predicted were under significant positive pressure and were investigated for frequency both before and during the period of elevated case numbers (Supplementary Table S3).

## Results

### Genetic diversity of DENV1–4 genomes isolated in Nha Trang, Vietnam, between 2016 and 2019

**Figure 1.**
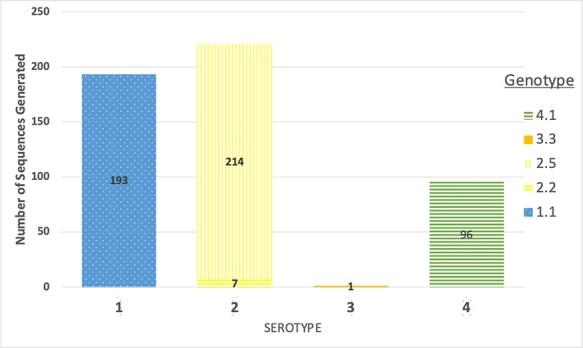
A stacked bar chart of the number of full DENV genome sequences generated during the study and their breakdown by serotype and genotype.

A total of 511 DENV full-length genomes were generated from the individuals sampled over the study period (2016–19). The breakdown of the serotype and genotype distribution of the sequences, as determined by the Genome Detective, is given in Fig. 1. Viruses from all four serotypes were observed, with a majority of D1 (37.8 per cent) and D2 (41.9 per cent) and fewer D4 (18.8 per cent) detected. D3 was represented by a single genome (0.2 per cent) and was excluded from subsequent analyses. Only one genotype within serotypes D1, D3, and D4 was identified (Genotypes I, III, and I, respectively), whilst, with serotype D2, sequences were of two distinct genotypes, namely II and V.

**Figure 2.**
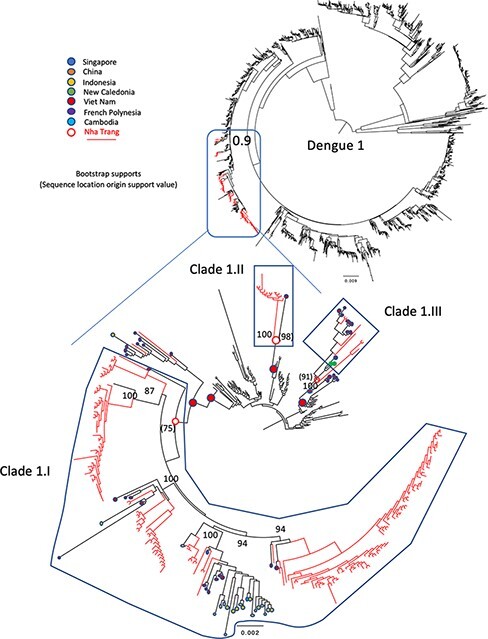
Midpoint-rooted maximum-likelihood trees of 2,446 publicly available full genome length DENV1 sequences (black terminal branches) with 193 DENV1 genomes generated in this study (red branches). The relevant region of the tree has been isolated as a separate tree. Branch supports, calculated as bootstrap scores from 1,000 replicates, are indicated on relevant branches. The sampling location of the publicly available sequences in a Nha Trang cluster is indicated by a coloured dot (see legend). The most likely country of origin of relevant internal nodes is indicated by a coloured node, with probability values given in brackets.

**Figure 3.**
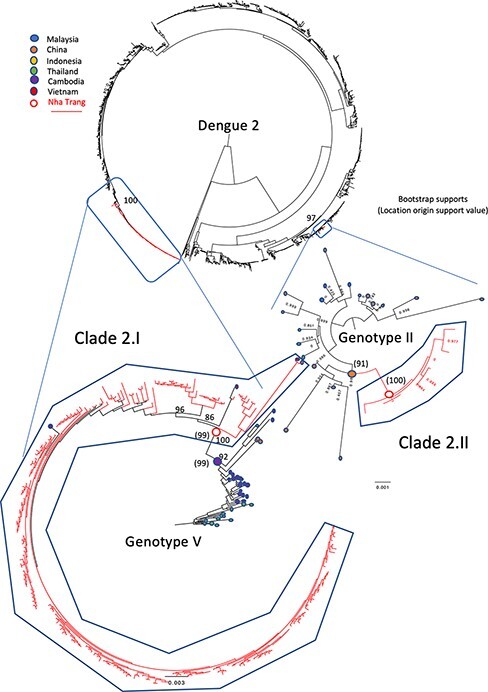
Midpoint-rooted maximum-likelihood trees of 1,891 publicly available full genome length sequences with the 221 full-length D2 genomes generated in this study shown as red branches. The relevant regions of the tree have been isolated as separate trees, with the difference in genotypes of these sequences shown. The given country of isolation for the most genetically similar corresponding sequences is marked as a coloured dot at the end of the branch. Bootstrap support values over 70 and on relevant supporting branches have been shown except for genotype II, which is small enough to show all bootstrap supports.

**Figure 4.**
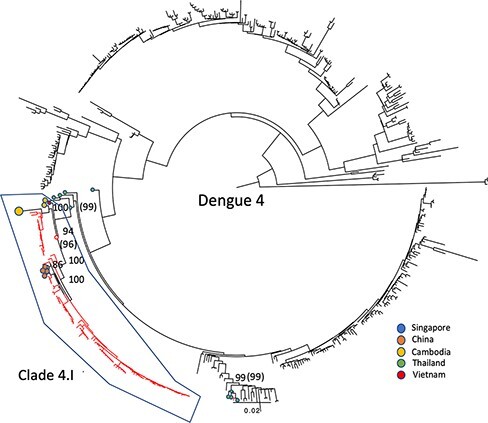
Midpoint-rooted maximum-likelihood trees of 407 publicly available full genome length sequences with the 96 D4 genomes generated in this study shown as red branches. The given country of isolation for the most genetically similar corresponding sequences is marked as a coloured dot at the end of the branch. Bootstrap support values over 70 and on relevant supporting branches have been shown.

ML phylogenies of the three studied serotypes combined with international full-length sequences available from GenBank confirmed the presence of multiple DENV lineages amongst the sampled infections (Figs 2**–**4). Clades were identified as clusters of sequences with the same geographical origin according to the mugration modelling in TreeTime and here are labelled according to their serotype and then given a clade number (e.g. ‘Clade 1.I’ is the largest clade identified for D1). The majority of the Nha Trang D1 genomes formed three distinct clades: (1) a large main cluster containing 173 local sequences (Clade 1.I in Fig. 2; 90 per cent of all Nha Trang D1 genomes) and 35 sequences from neighbouring countries and other parts of Vietnam (branch support 98.6 per cent), (2) a smaller cluster of thirteen sequences containing twelve local sequences and one from elsewhere in Vietnam (Clade 1.II in Fig. 2; support 100 per cent), and (3) a cluster mainly made of sequences from southern Vietnam and neighbouring countries (*n* = 17) but also including seven sequences from Nha Trang (Clade 1.III in Fig. 2; branch support 100 per cent). These findings suggested multiple introductions of DENV D1 into Nha Trang prior to the commencement of the study and over the course of the 3 years of the sampling in our study.

The D2 phylogeny also indicated at least two introductions of this serotype in the area (Fig. 3), with one large clade of 218 genotype V sequences, including 214 from this study in Nha Trang and 4 from southern Vietnam, China, and Cambodia (Clade 2.I in Fig. 3; 100 per cent branch support), and a second smaller clade (Clade 2.II; branch support 100 per cent) of 7 genotype II genomes from Nha Trang.

All but one D4 genome formed one large clade of 101 sequences (Clade 4.I in Fig. 4; branch support 96 per cent) and included sequences from China, Cambodia, and elsewhere in Vietnam. The remaining D4 genome clustered distinctly with sequences isolated from Thailand (branch support 99 per cent).

At least one large clade of Nha Trang sequences (i.e. Clades 1.I, 2.I, and 4.I) was observed in each of the phylogenies (Figs 2**–**4), suggesting that, for each serotype, a vast majority of the studied cases were sampled within local transmission chains. The average number of nucleotide substitutions per site within each clade is listed in Supplementary Table S1 and depicted in Supplementary Figure S1.

Isolated sequences (sequences with no close relative from the study and also in the phylogeny) were observed for serotypes D1 and D4, indicating independent introductions that either did not spread in the population or, more likely, were not sampled. Conversely, we observed that D1 and D2 sequences clustered very closely with other sequences isolated from Nha Trang and Vietnam, whilst there were far fewer D4 sequences available; consequently, clustering was limited to a group of sequences imported into China.

### Evidence for DENV persistence in the study area

**Figure 5.**
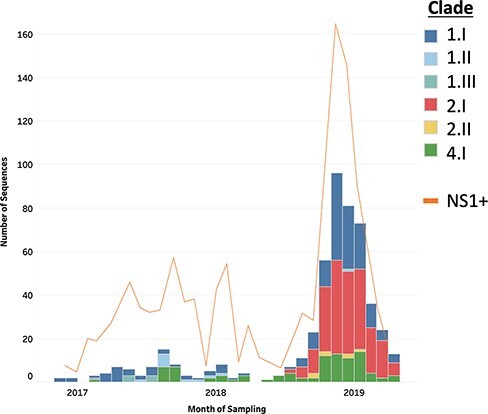
The number of sequences in each clade sampled each month from October 2016 to April 2019 with a total number of NS1-positive individuals recorded during the same time period.

The sampling time interval of the viral genomes within each local clade indicated that these chains of transmission persisted for between 121 days (Clade 2.II) and 878 days (Clade 1.I) during the sampling period in the area (Fig. 5). This provided a complex picture of serotype prevalence shifting over time, but with at least two distinct serotypes present every month of the sampling interval from April 2017 onwards. Serotypes D1 and D4 were ubiquitous and remained in the population for at least 2 years despite periods of low incidence and even after the introduction of D2 (first sample: August 2018) and the resulting outbreak of 2018–19. Our sampling strategy did not change during the sampling period, suggesting that it was the increase in reporting dengue cases during the outbreak that allowed more samples to be collected, which led to the consequent increased sensitivity to detect more clades during this period. This absence of strain replacement and long-term persistence of multiple serotypes in the population in the local area suggest sufficient levels of susceptible hosts and ongoing transmission to maintain the lineage all year round, even between DENV outbreak periods, when incidence is low, and despite vector control in the affected areas.

Ancestral state reconstruction revealed that, of the six clades identified in Nha Trang across the three serotypes, four had an ancestral node inferred to be located in Nha Trang with a probability greater than 75 per cent: Clade 1.I, Clade 1.II, Clade 2.I, and Clade 4.I. These clades were considered to represent local transmission chains and were further analysed. Inversely, the most likely place of origin of Clade 2.II was estimated to be China (Fig. 3; probability 91 per cent) and was excluded from the persistence time quantification.

**Figure 6.**
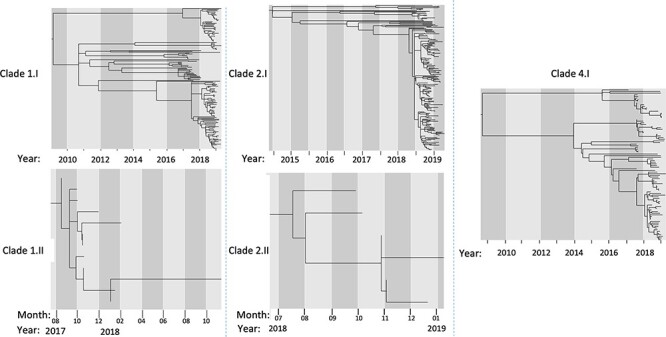
Dated phylogenies of five DENV clades sampled in Nha Trang between 2016 and 2019. Branch lengths correspond to time, with the year and month of the node shown in x-axis.

The complete time of persistence of these local transmission clusters was estimated from dated phylogenies as the time span between the estimated time of introduction of a clade’s founder virus in the area and the time of sampling of the most recent isolate in that cluster (Fig. 6), using the sampling dates of the Nha Trang genomes to calibrate the molecular clock.

**Figure 7.**
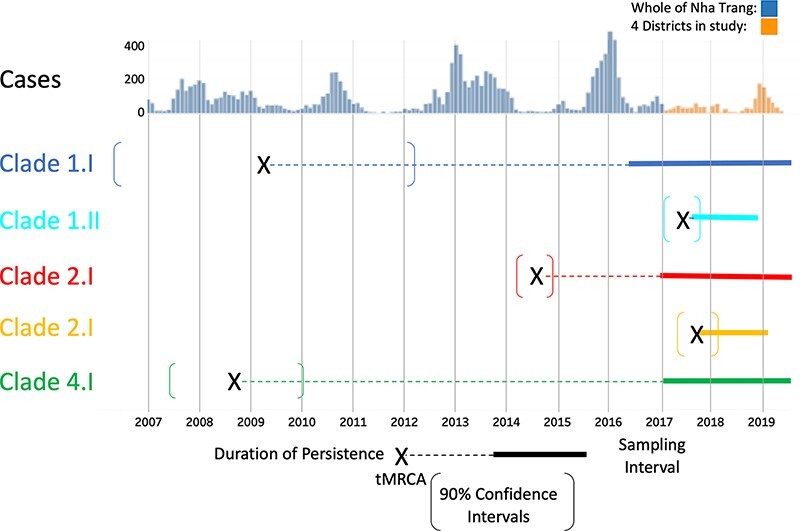
The timescale of sampled and predicted historical samples based on linear regression analysis of phylogenetic diversity overlayed on historical and recently reported DENV cases in Nha Trang. Sequences have been divided into serotypes and then coloured by clade according to genetic similarity and putative node origin. Fixed lines denote that a sequence has been sampled from at least a 2-month time period, whilst dotted lines are the hypothesised TMRCA denoted as an X with 90 per cent confidence intervals denoted by square brackets.

Calculating the TMRCA of the identified clades suggested that the first of the clades to be imported into Nha Trang was Clade 4.I, with a date of introduction as early as September 2008 (90 per cent CI: May 2007 to January 2010), followed by Clade 1.I. (March 2009; 90 per cent CI: July 2006 to November 2011), Clade 2.I (June 2014; 90 per cent CI: July 2012 to May 2015), Clade 1.II (June 2017; 90 per cent CI: February 2017 to August 2017), and Clade 2.II (June 2018; 90 per cent CI: March 2018 to September 2018) (Fig. 7 and Supplementary Table S2). Two of the clades showed evidence of at least one decade of local persistence, with intervals of 3,887 days (Clade 4.I) and 3,707 days (Clade 1.I) between the estimated time of introduction and the most recent sampling. We see introduction events of novel clades (Clades 1.II and 2.II) to the population during a low case number season; these remained and were observed during the peak outbreak season in 2019. No introduction events were noted during or within the previous year of 2019.

## Discussion

This study provides strong evidence of the persistence of locally circulating DENV strains throughout its almost 3-year duration, determined using phylogenetic techniques. This is despite significant fluctuations in case numbers and control measures implemented by the Vietnam National Dengue control programme (World Health Organization 2017). In addition to the continued prevalence of the clades that were directly sampled, we also estimated that two of these circulating lineages had likely been present in the area for a decade. Introduction events of new viral lineages that were distinct from those in persistent circulation were also observed during the study period.

Categorising clades according to the phylogenetic similarity of the sampled viral sequences showed that Clades 1.I and 4.I persisted for the longest time period, whilst the more recently introduced Clade 2.I became the dominant clade sampled during the final year of our study. In the final year, an increase in case numbers compared to other years occurred that coincided with the rainy season in this area of Vietnam (Cuong et al. 2013) although interestingly all recent putative introduction events appeared to occur outside of these peak years. This was a pattern that was also reported across the whole of Vietnam and conforms to the cyclic nature of DENV infections that has been reported elsewhere, with cases peaking roughly every 3 years (Cummings et al. 2004; World Health Organization – Western Pacific Region 2019). Our results suggest that some of these recent increases in cases may have been related to the recent introduction of two new D2 clades into the population (Clades 2.I and 2.II) although further serology work would be required to confirm this effect, and the effects of increased and improved sampling and diagnosis cannot be excluded.

The successful establishment of a new viral lineage has been suggested to be dependent on the historical exposure to different DENV serotypes of a population, potentially generating an immunological niche of more susceptible individuals to the new serotype, with previous studies showing that novel serotype introductions into a naive population are more likely to become established and cause greater case numbers than those for which there is already a serotype in long-term circulation (Sim and Hibberd 2016). For the establishment of viral lineages of a similar serotype to historical exposures, previous investigations have suggested that positive selection for epidemic potential may have occurred, which could potentially explain the increase in case numbers. In our analysis of both pervasive and episodic changes in the genome, we detected a mixed picture (Supplementary Table S3), with Clade 1.1 showing some evidence of selection associated with the later increase in numbers, while Clade 4.1 did not. Given that the numbers of all three main clades were observed to increase simultaneously during this later period, it seems likely that environmental factors, such as the number of mosquitoes, were more important than virological adaptation.

The low case numbers sampled at certain times of the year, with prolonged periods of weeks and even months where no clade members were sampled, could reflect successful control strategies. However, phylogenetic inference has now enabled us to show the subsequent re-emergence of the same clade at later dates, with the expected low number of observed mutations accumulated during the missing time period. The virus therefore continues to be cryptically transmitted in the population and is likely not detected due to a lack of sampling during this period. Sampling is generally lower during periods that also coincide with unfavourable conditions for the vector (Lee et al. 2017). Such a period of low transmission, aggravated by a high prevalence of underlying asymptomatic and mildly symptomatic transmission (estimated to compromise up to 84 per cent of total DENV infections in a population (Ten Bosch et al. 2018)), is suggestive of unsampled subclinical infection as the most likely explanation for viral persistence of these clades.

Our data also provide evidence for viral persistence despite attempts of local control although continued reimportation of the same viral clade, probably from nearby areas, is also possible. We suspect this local reimportation may have happened with Clades 1.I and 4.I, where we see distant ancestral branches between subclades (Fig. 6). This implies missed sampling in the local area over the many years since the predicted TMRCA of these clades (Fig. 5). This highlights one of the limitations with this study where incomplete sampling of the total infected population will likely cause us to not fully capture the total viral diversity of each clade, which can cause an underestimation of the calculated TMRCAs and in turn of the time of introduction of the observed lineages. In order to mitigate this lack of sampling, we included all available sequences from GenBank, which included previously collected samples from Nha Trang and elsewhere in Vietnam. However, this did not alter our findings, and we were still able to observe prolonged periods of persistence in the population.

These findings have implications for future control strategies for DENV, as we have shown that there is continued, often unreported DENV transmission year-round. Vector control could therefore be implemented all year, as well as during peak periods as is currently favoured (Nguyen-Tien, Probandari, and Ahmad 2019). The cyclic nature of DENV case numbers, with peak outbreak periods occurring approximately every 3 years in this population (Quyen et al. 2018), indicates a role for increased surveillance and control measures during outbreak years and potentially the use of vaccines or prophylaxis if available (Kaptein et al. 2021). The discovery of imported cases into the area from other regions of Vietnam as well as from abroad, one of which rapidly became the dominant circulating lineage, highlights a potential role of border sampling of international visitors, particularly from DENV endemic regions with a high prevalence of unseen DENV serotypes (such as D3 of which very few cases were observed in this study period).

## Conclusions

We have identified epidemiological and phylogenetic evidence of uninterrupted transmission chains that have lasted for the 3 years of our sampling period, and through the use of TreeTime, we have inferred that some of these transmission chains may have persisted for more than 10 years. During this persistence, infected cases per year have increased and decreased, implying that environmental changes (which highly influence mosquito numbers) are more likely to be the cause of observed changes in human infections rather than the introduction of clades with specific viral adaptations. In addition, our data also imply that anti-dengue strategies had only limited success in preventing transmissions, even in the low seasons or non-outbreak years, possibly due to the importance of unreported (potentially sub-symptomatic) cases.

## Supplementary Material

vead012_Supp

## Data Availability

Processed FASTA files used in this study are availble upon request and have been deposited at the National Center for Biotechnology Information (NCBI OQ426566–OQ427062).
