# Supplementary material for: A phylogenetic study of dengue virus in urban Vietnam shows long-term persistence of endemic strains
Source: Virus Evol. 2023 Feb 16;9(1):vead012. doi: 10.1093/ve/vead012 (PMC10013730; doi:10.1093/ve/vead012)
Supplement: vead012_Supp [file vead012_supp.zip › Supplementary Table 1.docx]

Supplementary Table 1: Supplementary Table 1: Distribution of average p-distances among sampled clades

| Clade | Size | Nucleotide substitutions/site (x10^-3^) | Standard Error  (x10^-3^) | Genome Length (base pairs) | Average mutation sites/genome |  |
| --- | --- | --- | --- | --- | --- | --- |
| 1.1 | 173 | 9.68 | 0.52 | 10415 | 100.85 |  |
| 1.2 | 12 | 0.44 | 0.098 | 10415 | 4.57 |  |
| 2.1 | 214 | 2.46 | 0.18 | 10411 | 25.61 |  |
| 2.2 | 7 | 9.68 | 0.52 | 10411 | 100.81 |  |
| 4.1 | 95 | 9.80 | 0.49 | 10594 | 103.83 |  |
