# Supplementary material for: A phylogenetic study of dengue virus in urban Vietnam shows long-term persistence of endemic strains
Source: Virus Evol. 2023 Feb 16;9(1):vead012. doi: 10.1093/ve/vead012 (PMC10013730; doi:10.1093/ve/vead012)
Supplement: vead012_Supp [file vead012_supp.zip › Supplementary Table 2.docx]

Supplementary Table 2: Summary of clade persistence findings from both sampling data and estimated tMRCA inference

| **Serotype** | **Clade** | **Sampling dates** | | | **tMRCA (90% CI)** | | | |
| --- | --- | --- | --- | --- | --- | --- | --- | --- |
|  |  | First | Last | Duration  (days) | Estimated root date | Min | Max | Duration  (days) |
| 1 | I | 28/11/2016 | 25/04/2019 | 878 | 03/2009 | 07/2006 | 11/2011 | 3707 |
|  | II | 15/06/2017 | 10/12/2018 | 543 | 06/2017 | 02/2017 | 08/2017 | 557 |
| 2 | I | 19/07/2018 | 26/04/2019 | 281 | 06/2014 | 07/2013 | 05/2015 | 1790 |
|  | II | 10/09/2018 | 09/01/2019 | 121 | 06/2018 | 03/2018 | 09/2018 | 222 |
| 4 | I | 17/02/2017 | 24/04/2019 | 796 | 09/2008 | 05/2007 | 01/2010 | 3887 |
