# Supplementary material for: A phylogenetic study of dengue virus in urban Vietnam shows long-term persistence of endemic strains
Source: Virus Evol. 2023 Feb 16;9(1):vead012. doi: 10.1093/ve/vead012 (PMC10013730; doi:10.1093/ve/vead012)
Supplement: vead012_Supp [file vead012_supp.zip › Supplementary Table 3.docx]

Supplementary Table 3: Positive selection analysis of clades which spanned the full study period. MEME and FUBAR analysis have been performed with sites with significance values less than 0.05 and greater than 0.9 shown. The distribution of amino acids before and after the elevated case number period are shown.

| Clade | AA location | Viral protein | MEME (p-value) | FUBAR (probability) | Percentage before elevated cases | Percentage during elevated cases |
| --- | --- | --- | --- | --- | --- | --- |
| 1.I | 183 | Membrane Glycoprotein Precurser | NO | YES (0.978) | 100% Alanine | 37.3% Alanine : 62.7% Threonine |
| 1.I | 878 | NS1 | NO | YES (0.961) | 100% Isoleucine | 63.2% Isoleucine : 36.8% Valine |
| 1.I | 375 | Envelope | YES (0.01) | YES (0.931) | 61.1% Phenylalanine : 38.9% Leucine | 100% Phenylalanine |
| 4.I | 3164 | NS5 | YES (0.01) | YES (0.96) | 83.3% Cysteine : 16.7% Histidine | 100% Cysteine |
| 4.I | 174 | prM | YES (0.03) | NO | 100% Threonine | 97.2% Threonine : 2.8% Isoleucine |
| 4.I | 670 | Envelope | YES (0.04) | NO | 95.8% Threonine : 4.2% Serine | 98.6% Threonine : 1.4% Isoleucine |
