# Supplementary figures and images for: A phylogenetic study of dengue virus in urban Vietnam shows long-term persistence of endemic strains
Source: Virus Evol. 2023 Feb 16;9(1):vead012. doi: 10.1093/ve/vead012 (PMC10013730; doi:10.1093/ve/vead012)

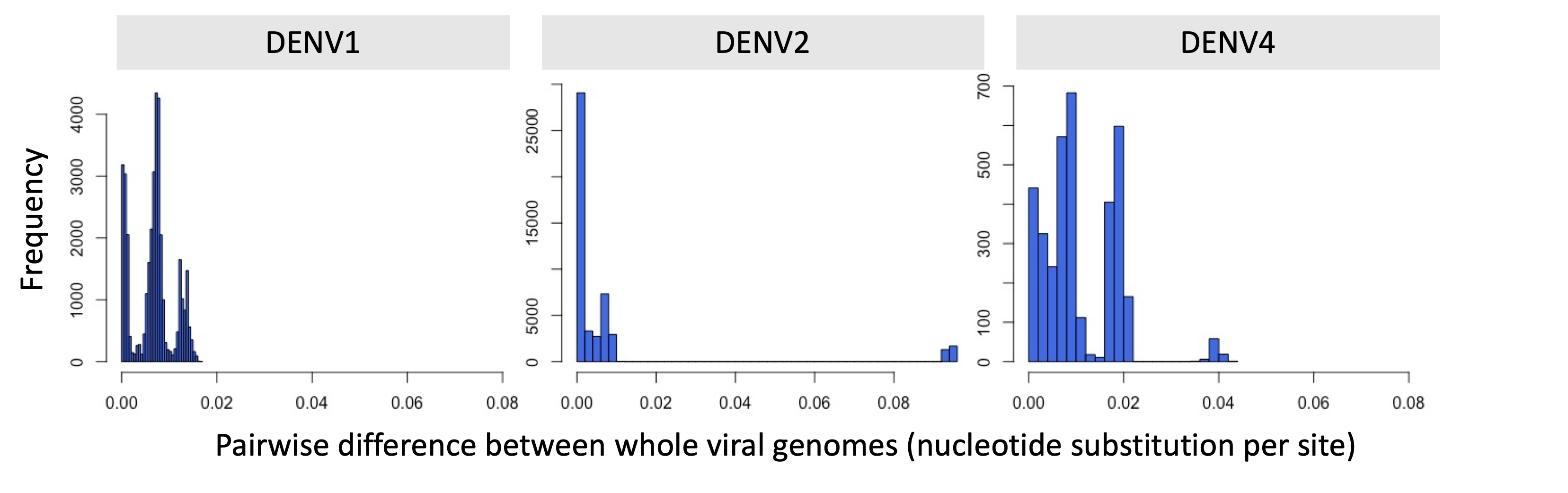

Supplement: vead012_Supp [file vead012_supp.zip › supfig1.tiff]
